# Digital advance care planning with severe mental illness: a retrospective observational cohort analysis of the use of an electronic palliative care coordination system

**DOI:** 10.1186/s12904-024-01381-y

**Published:** 2024-02-26

**Authors:** Rea Kaur Gill, Joanne Droney, Gareth Owen, Julia Riley, Lucy Stephenson

**Affiliations:** 1 Institute of Psychiatry, Psychology and Neuroscience, King’s College London, 16 De Crespigny Park, London, SE5 8AB UK; 2 The Royal Marsden, Fulham Road, London, SW3 6JJ UK; 3 Imperial College London, Exhibition Road South Kensington, London, SW7 2BX UK; 4 South Central and West Commissioning Support, 18-20 Massett Road, Horley, RH6 7DE UK

**Keywords:** Severe mental illness, Palliative care, Advance directive, Advance decision, Digital systems

## Abstract

**Background:**

People living with severe mental illness (SMI) face significant health inequalities, including in palliative care. Advance Care Planning (ACP) is widely recommended by palliative care experts and could reduce inequalities. However, implementing ACP with this group is challenging. Electronic Palliative Care Coordination Systems such as Coordinate my Care (CMC) have been introduced to support documentation and sharing of ACP records with relevant healthcare providers. This study explores the use of CMC amongst those with SMI and aims to describe how those with a primary diagnosis of SMI who have used CMC for ACP, and makes recommendations for future research and policy.

**Method:**

A retrospective observational cohort analysis was completed of CMC records created 01/01/2010–31/09/2021 where the service user had a primary diagnosis of SMI, with no exclusions based on comorbidities. Descriptive statistics were used to report on characteristics including: age, diagnosis, individual prognosis and resuscitation status. Thematic analysis was used to report on the content of patients’ statements of preference.

**Results:**

1826 records were identified. Of this sample most (60.1%) had capacity to make treatment decisions, 47.8% were aged under 70, 86.7% were given a prognosis of ‘years’ and most (63.1%) remained for full cardio-pulmonary resuscitation in the event of cardio-pulmonary arrest. Records with completed statements of preferences (20.3%) contained information about preferences for physical and mental health treatment care as well as information about patient presentation and capacity, although most were brief and lacked expression of patient voice.

**Discussion:**

Compared to usual CMC users, the cohort of interest are relatively able, younger people using CMC to make long-term plans for active physical and mental health treatment. ADM is a service user-driven process, and so it was expected that authentic patient voice would be expressed within statements of preference, however this was mostly not achieved.

**Conclusions:**

This digital tool is being used by people with SMI but to plan for more than palliative care. This cohort and supporting professionals have used CMC to plan for longer term physical and mental healthcare. Future research and policy should focus on development of tailored digital tools for people with SMI to plan for palliative, physical and mental healthcare and support expression of patient voice.

## Introduction

People living with severe mental illness (SMI) experience significant health inequalities. They have a higher risk of poor physical health compounded by poor access to care [1–3]. Research shows this adds up to a mortality gap of 10–20 years reduction in life expectancy [4]. This gap is likely to be due to an increased vulnerability to chronic physical disease such as obesity, diabetes, cardiovascular disease and cancer plus side effects of psychiatric medication, higher rates of unnatural death and increased barriers to accessing physical health care [5, 6]. This inequality extends to palliative care. People living with SMI can be understood as at risk of ‘disadvantaged dying’ [7]. They are more likely to present to services at a late stage of a physical illness, with complex co-morbidities and experience stigma and poor collaboration between physical and mental health services [8]. So far, the research on interventions which may help improve their experience has been limited [9].

Advance Care Planning (ACP) is used throughout this paper as an umbrella term which refers to planning for future care. It can involve making legally meaningful advance decisions (e.g. Advance Decisions to Refuse Treatment and Advance Statements under the Mental Capacity Act 2005) and commonly infers Shared Decision Making between health professionals and patients/service users. ACP has been widely embraced within the palliative care community [10] and is included in NICE guidelines [11]. It has been shown, when implemented fully and with buy-in from service users and healthcare professionals, to improve communication, personalised care and quality of life within different patient groups [12, 13]. It has also been identified as a tool which has potential to reduce inequalities experienced in mental health [14, 15] and palliative care [8].

However, implementing ACP with this population can be challenging and people with SMI are less likely to be engaged in ACP around palliative care [16] This may be because professionals are concerned about causing distress in a vulnerable population and may assume they lack capacity to make these kinds of decisions [8]. Research with service users challenges these assumptions and has found that they do wish to engage in decision making on their physical health care and with additional decision making support they do have the capacity to make these decisions [17, 18].

### Digital advance care planning records and SMI

One approach that has been developed to support palliative ACP in the general population is the use of Electronic palliative care coordination systems (EPaCCS). These systems were developed across UK to support documentation and sharing of clinical information and patient-specific priorities for care in order to facilitate communication and enhance patient-centered care for patients at end of life [19]. Nationally, at least 175 (83%) clinical commissioning groups (CCGs) (now reorganised as Integrated Care Boards) have either implemented EPaCCS or started planning for their implementation [20].

The aim of this study is to explore the use of one EPaCCS known as ‘Coordinate My Care’ (CMC) as a digital service to record advance care plans with individuals living with SMI.

1. To analyse key demographic features of patients with a primary diagnosis of SMI who use CMC, e.g., age, gender, comorbidities, expected prognosis.
2. To describe how the digital service CMC is utilised when a patient has a primary diagnosis of SMI.
3. To consider the implications of CMC use by people with SMI on policy and practice.

## Methods

### Design

This was a retrospective observational cohort study of anonymised Coordinate My Care (CMC) records. CMC was commissioned as the EPaCCS in London between 2010 and March 2022, during which time over 144,000 patient plans were created. It functioned as a digital NHS care planning and communication service, for use by patients, clinicians, and practitioners to document and share demographic and clinical information, advance decisions and priorities and preferences for treatment and care with all relevant healthcare providers. Patient preferences about end-of-life care are recorded on CMC, including preferred place of care and death. Clinical decisions about cardiopulmonary resuscitation status and ceiling of treatment are also recorded on the CMC plan by the patient’s healthcare professional, to inform clinical treatment in the event of deterioration. A “ceiling of treatment” (also known as a Treatment Escalation Plan) is the clinical decision about the appropriate level of clinical intervention for an individual patient based on their previous and current medical needs [21]. CMC plans could be updated and viewed in real time by hospitals, emergency care services, hospices, care homes and primary care practitioners. CMC plans were jointly created by clinicians with patients and their families. Patients, if they wished, could start creating their own CMC plans (via MyCMC which was introduced in May 2019) where they could enter their own personal data and information related to care preferences and wishes. Their doctor or nurse could then add the clinical data, confirm the plan with the patient/family and make the CMC plan live on the CMC system. Capacity statements (whether the patient does/does not have capacity) for certain decisions (e.g. creating a CMC record, CPR decisions) are inbuilt within the CMC record and as such shared with relevant healthcare providers to inform treatment in the event of a clinical deterioration.

If the patient does not have capacity to consent themselves, a CMC record can be created for them in their best interest by their clinical team, sometimes with input from a Lasting Power of Attorney. By using CMC, patients are involved in their own decision-making and care, reduce their frequency of unwanted hospital admissions, and have a plan that all healthcare professionals involved in their care can access and update [22].

### Participants

Patients aged 18 and over who created a CMC plan between 01/01/2010 and 31/09/2021, who had a primary diagnosis of SMI were included in this analysis. SMI diagnosis included major depression, bipolar disorder (ICD-10 mood (affective) disorders: F30-39) and schizophrenia (ICD 10 schizophrenia, schizotypal and delusional disorders: (F20-29). CMC records for those without a primary diagnosis of a mental health disorders within these ICD-10 categories were not extracted and therefore excluded from this sample.

### Procedure

Anonymised patient-level data were extracted and analysed using descriptive statistics. The demographics of the patients with CMC records were explored in terms of age, gender, comorbidities and each individual’s documented prognosis. The content of CMC plans for people with severe mental illness was examined to describe the elements of advance care planning for end of life care that were completed: resuscitation status, ceiling of treatment, and preferred place of death. Ceiling of treatment refers to the maximum level of intervention a patient wishes to have if they fall ill. For example, they may wish to have full treatment of any illness or just medication to be kept comfortable without treating the cause. The analyses were carried out on the whole study cohort and also stratified by age (under 70 years old compared to over 70 years old), individuals’ prognosis (short-term i.e., days to months, compared to long-term i.e., years) and outcome at time of analysis (deceased compared to non-deceased). Thematic analysis was also employed to analyse the content of the ‘patient wishes’ free text field, which was an optional field for patients and clinicians to include anything else deemed important to them.

## Results

There were 1826 patient cases between 01/01/2010 and 31/09/2021 who had a primary diagnosis of severe mental illness. Of this sample, 55.7% (*n* = 1018) were female, 52.2% (*n* = 953) were aged 70 or over at plan creation, 86.7% (*n* = 1605) had a prognosis of ‘years’, and 61.2% (*n* = 1118) had a primary diagnosis of schizophrenia (ICD-10 F20). 61.2% (*n* = 1097) were assessed to have capacity to consent to their plans, 36.9% (*n* = 674) had a DNACPR in place, 31.8% (*n* = 580) listed a care home as their first-choice preferred place of death, 53.1% of the plans in this sample were created by a GP, and, for those who had died, there was an average of 503 days between plan creation and death (minimum=-295, maximum = 3301). Most of the group had another secondary diagnosis, with 62.7% (*n* = 1145) having a physical comorbidity. These demographics are described in Table 1 with plan details described in Table 2.

**Table 1:** Baseline demographics of sample

	(%)	(n)
Gender		
Female	55.8	1018
Male	44.2	808
Age at plan creation		
18–39	6.6	120
40–49	6.5	119
50–59	14.9	272
60–69	19.8	362
70–79	26.7	488
80+	25.5	465
Primary Diagnosis		
Schizophrenia	61.2	1118
Depression	24.2	442
Bipolar Disorder	14.6	266
Secondary Diagnosis		
Physical health comorbidity	62.7	1145
Mental health comorbidity	7.7	141
No secondary diagnosis	29.5	539
Documented Prognosis		
Days	0.7	13
Weeks	2.3	42
Months	7.0	128
Years	87.9	1605
Capacity to consent to plan		
Yes	60.1	1097
Patient lacking capacity to created CMC plan: CMC plan created in the patient’s Best interests	35.2	642
Patient lacking capacity to create CMC plan: CMC plan created with input from Lasting Power of Attorney	4.8	87

**Table 2:** Coordinate my care patient plan details

	(%)	(n)
CPR decision		
For cardio-pulmonary resuscitation	63.1	1152
Not for cardiopulmonary resuscitation	36.9	674
Ceiling of Treatment		
Full active treatment – including CPR	59.4	1084
Full active treatment – not including CPR	6.8	125
Treatment of reversible physical conditions only, including hospital	16.2	295
Treatment of reversible physical conditions only, not hospital	9.3	170
Symptomatic only, keep comfortable	3.9	72
Preferred place of death – First choice		
Care home	31.8	580
Hospital	6.4	116
Hospice	0.9	16
Home	13.6	248
Community Hospital	0.3	6
Other	3.7	67
None recorded	43.4	793
Preferred place of death – Second choice		
Care home	11.3	207
Hospital	9.3	170
Hospice	2.6	48
Home	3.7	68
Community Hospital	0.5	9
Other	2.8	51
None recorded	69.7	1273

### Old vs. young

Of the sample, 47.8% (*n* = 873) were younger (aged 18–69) and 52.2% (*n* = 953) were older (aged 70+). 69.3% (*n* = 605) of the young group had capacity to consent to their plan compared to 51.6% (*n* = 492) of the older group. Of the young group, 94.2% (*n* = 822) were given a long-term prognosis, compared to 82.2% (*n* = 783) of the older group. Just 12.7% (*n* = 111) of the young group had a DNACPR in place compared to 59.1% (*n* = 563) of the older group. When looking at those aged 80 and over, 72.5% (*n* = 337) had a DNACPR in place.

### Short-term vs. longer-term prognosis

In this sample, only 10.0% (*n* = 183) were given a short-term prognosis (days, weeks, months) compared to 87.9% (*n* = 1605) given a longer-term prognosis (year, years), with 2.0% (*n* = 38) undefined. Of those given a longer-term prognosis, 62.6% (*n* = 1005) had capacity to consent, as opposed to just 36.1% (*n* = 66) of those given a short-term prognosis. 31.3% (*n* = 503) of those given a longer-term prognosis had a DNACPR in place, compared to 88.5% (*n* = 162) of those given a short-term prognosis. 47.0% (*n* = 754) of those given a longer-term prognosis did not record a preferred place of death, in contrast with just 18.0% (*n* = 33) of those given a short-term prognosis.

### Capacity vs. without capacity

60.1% (*n* = 1097) of the sample had capacity to consent to the creation of the CMC plan. Of those, 91.6% (*n* = 1005) were given a longer-term prognosis, compared to 82.3% (*n* = 600) of those without capacity. Just 26.2% (*n* = 287) of those with capacity had a DNACPR in place, as opposed to 53.1% (*n* = 387) of those without capacity. Of those with capacity, 47.5% (*n* = 521) did not have a recorded preference for place of death, compared to 37.3% (*n* = 272) of those without capacity.

### Patient wishes

Inductive thematic analysis was used to analyse data from the ‘patient wishes’ free-text column (n = 371). Thematic analysis was chosen as it is an accessible, flexible method of analysing participant-generated textual data [23]. A variety of approaches were taken to completing this column. These included professionals completing an entry entirely on behalf of the patient using medical terminology, professionals recording their views of the patient’s wishes and the patient’s wish recorded verbatim in a structured or unstructured format. Many responses were short-form statements around patient capacity (“patient does not have capacity to discuss”) or specific (“full active treatment, not CPR”), with limited expression of patient views. Some responses followed a more structured template, outlining patients’ likes/dislikes, thoughts about their wellbeing, and preferences around specific treatments. Although these responses were in the minority, they contained the most insight and were written in the first person, thus suggesting that they were written by the patient themselves using the online MyCMC portal.

There were four main themes identified in the ‘patient wishes’ data: (1) expressing preference for treatment, (2) expressing preference for care, (3) stating patient presentation, and (4) stating patient capacity.

### Theme 1: expressing preferences for treatment

There were three sub-themes identified: expressing preferences around (1) physical health treatment, (2) end-of-life treatment and (3) mental health treatment. The majority of responses in this theme related to the physical health treatment sub-theme, namely detailing a preference for full active treatment although some responses were around specific treatments and medications, such as “doesn’t want blood transfusions” and “antibiotics as a last resort only for reversible conditions”. A portion of responses were expressing preferences around end-of-life treatments, mainly around whether the person would pr would not like resuscitation, stating either “does not wish to be resuscitated” or “[patient} expressed her wish to be resuscitated”. The responses about mental health treatment mainly revolved around medication preferences during inpatient treatment. This is illustrated by one person who stated:

My preferences for mental health medical treatments: Have one Valium or sleeping tablet in the house at all times on top of the sodium valproate in case I stop sleeping to help me catch it early. Sometimes they have to inject me to get to sleep and I don’t want it to get to that stage. I would rather take Valium than Olanzapine if I can’t sleep.

### Theme 2: expressing preferences for care

There were two sub-themes identified: expressing preferences for (1) physical health care (2) end-of-life care (3) mental health care. Responses discussing physical health care often detailed patients not wanting to be admitted to hospital unless necessary, if at all. They included information about care preferences from staff and family involvement, such as “[patient] hates going to hospital often refuses to go has a good relationship with his carer wishes to remain at home”. Another person stated “in case someone else have to complete my personal care for me it doesn’t matter the gender, and I would like to have it done every day”. Responses around end-of-life care followed a similar trend of patients not wanting to be admitted to hospital unless necessary such as “patient verbalised her wishes to die at home”. Some included information about post death care; funeral, burial arrangements and family involvement. One record stated “[patient] wants to be where his parents are in crematorium in [location]”. Preferences for mental health care paralleled the themes around physical health care and included views around the need to go to hospital, as illustrated by one person who said “if I get to the point when I am not listening to anyone it is an outrage that I cannot go into hospital straight away”. Responses also included the and the type of support that might be required from professionals, family and friends when distressed, such as stating “I am physically well but mentally I continue to suffer anxiety when I am distressed. I keep whaling [wailing] and staff/family/friends have to reassure me”. Another person stated “I find my social life helps with any low periods connected with bipolar – without the right medication, activities, and socialisation, my bipolar could become a more prevalent and serious issue”. However, many entries were so brief that it was challenging to differentiate whether physical or mental health care needs were being considered.

### Theme 3: stating patient presentation

A large portion of responses used the ‘patient wishes’ textbox to state the current presentation of the patient. Many of these responses appeared to follow a template that asked patients to state their thoughts about their current health and wellbeing, such as “My thoughts about my health and wellbeing – I believe that I am feeling good”. Another example of this is “My thoughts about my health and wellbeing – I have physical and mental health problems. I do suffer from paranoid schizophrenia and diabetes and struggle too [sic] manage it without support”. Some responses indicated consideration of future presentation, such as “I have good general health enabling me to live independently. Illness may require me to move into a care home”. Other responses indicated that a medical professional had completed the plan on behalf of the patient, for example “patient’s baseline – mobility but requires all prompting and personal care from staff/family/friends. She can be aggressive and shout at times. High falls risk”. Another response stated “thought he was in hospital and wanted to go and see his mum who died many years ago”.

### Theme 4: stating patient capacity

In some cases, this field was used to re-iterate that the individual patient did not having capacity to engage in discussions around their care. The majority of responses were short, stating “lacks capacity to discuss” or “unable to discuss”, however some responses provided some further detail around patient capacity. For example, one response stated “unable to discuss with resident. CMC created in best interests after d/w [discussion with] GP, Dr X. Fluctuating mental capacity”. One specific response provided historical wishes of the patient, despite a lack of current capacity:

[Patient wishes] not known. Patient advised by care home manager as not having capacity to make decisions about his future care. Unfortunately, there is no record of any future wishes when he had mental capacity to make them. From previous discussions however, the care home manager is aware he does not like hospitals and had resisted admissions as much as possible.

## Discussion

### Summary of key findings

The current study aimed to evaluate how a digital tool supporting advance care planning in palliative care settings is used by people with SMI. This is important to understand as this population is vulnerable to experiencing ‘disadvantaged dying’ [7]. Engagement with ACP could address some of these disadvantages, however, previous research demonstrates it can be more difficult for people with SMI to create ACP documents which plan for palliative care [8]. The study showed that there is some engagement of CMC use amongst this population with 1826 digital ACP records (out of over 144,000) documents created using the system. However, an important finding is that this cohort had some unexpected demographic characteristics. A significant proportion (37.3%) did not have physical comorbidities and were younger (47.8% under 70). Most had the capacity to make decisions about care and treatment (60.1%), were not at the end of life (87.9% prognosis of years) and were requesting active treatment (63.1% for CPR). Results from the qualitative analysis illuminates how this digital tool is being used by people with SMI and professionals supporting them. Free text entry on patient wishes were largely unstructured and brief. Most did not record the patient’s preferences in their own words. Entries tended to include basic information on patient preferences for physical and mental health treatment and care, descriptive statements about presentation or information on mental capacity. These results suggest people with SMI and their supporting professionals are able to engage with CMC. These data do not however provide any information about whether or not patients found the process of creating or having a CMC plan useful or effective in terms of impact on care and treatment.

Further research is needed to examine the benefits of a digital service to support ACP in this complex and vulnerable population including impact on the management of mental health crises.

### Facilitating implementation of ADM documents for people with severe mental illness

Over the last two decades there has been increasing international interest in the use of ACP about treatment and care during mental health crises [14, 15]. Following this international trend, government in England and Wales have recently committed to incorporating statutory support for advance decisions in reforms to the Mental Health Act (1983). These will be known as ‘Advance Choice Documents’ and will be offered to all those who have previously be detained under the MHA [24].

During mental health crises people with SMI are at risk of experiencing compulsory treatment and detention and may lose the capacity to make treatment decisions. Making ACP documents when well may increase service user autonomy [14], increase therapeutic alliance [25] and reduce compulsory treatment [26, 27]. However, it is widely acknowledged that despite widespread service user interest uptake is limited [28, 29]. Barriers to uptake occur at systemic, service user and health professional levels [30] with health professional scepticism and lack of engagement identified as a central issue [31]. Some of this scepticism is fuelled by implementation concerns around lack of accessibility in a crisis rendering documents meaningless [32]. This study offers the following lessons to policy makers around implementing mental health ACP. Firstly, it suggests that clinicians do seem to be willing to engage with a *digital* tool which supports collaborative ACP for people with SMI despite the fact this tool is not yet tailored or promoted for this population. Therefore, it seems likely that a targeted digital product for mental health ACP may be highly relevant when seeking to implement government reforms to introduce Advance Choice Documents. Secondly, as previously discussed, a key barrier to ACP uptake has been identified as accessibility in a crisis. It is possible that the digital interconnectedness of CMC offers reassurance about this and hence more willingness to engage with the tool. Any future digital system should aim to replicate and tailor the digital availability that CMC offers. Thirdly, it is of note that 53.1% of CMC plans were created by a GP highlight the invaluable role of primary care in supporting patients with SMI to engage with ACP. GPs should be included in plans to train and resource health professionals around the introduction of mental health ACPs.

### Patient voice

Only a minority (371/1826) of plans made use of the free text to record patient wishes. In many records, documentation of in this section only contained clinical information about capacity and details of the patient’s presentation. The highest quality entries seemed to use a structured template to aid completion. This is consistent with work on computer aided mental health ADM creation tools which found that service users found structured prompts easier to work with than blank spaces [33]. The results also point to the need for specific training for health professionals on approaching and recording care planning discussions, this may help ensure entries are richer and more comprehensive. Studies on ACP in mental health have highlighted the importance of the role of an independent facilitator being involved in the process [34–36] to increase the uptake of mental health ACP and address power differentials. One study suggested that higher quality documents may be generated when a specially trained advocate supports document creation rather than clinicians [37]. Other models shown to be successful in RCTs and other research support a process of ACP which involves the service user, their loved ones, an independent facilitator and a health professional [35, 36]. This model could also be explored in palliative care settings with the SMI population to improve ACP quality. There are likely to be many factors underpinning engagement with CMC by this patient population. One may be the wish to be assured that healthcare professionals have access to up-to-date information about clinical treatment plans and patient care preferences, in line with “patient passports” which facilitate information sharing across different health organisations [38]. The unique benefit of CMC is that these data are digital, up to date and accessible in real time across healthcare providers and settings.

### Limitations

As this was a geographically limited sample, of which insufficient socioeconomic data was completed, the findings are difficult to generalise to the whole population of people with SMI. Consequently, this study should be considered exploratory. It would have been particularly helpful to have been able to explore the use of CMC according to ethnic grouping. This is because the ethnic disparities in receiving palliative care are widely acknowledged and people with SMI from Black backgrounds are likely to experience intersectional disadvantage [39]. Mental health ADM hold potential to be particularly important and most cost effective for people from Black communities living with SMI [40]. However, it is likely this group may experience additional barriers to completion [36].

There was evidence that people with SMI had utilised CMC for advance care planning, however, this group was only around 1.2% of approximately 144,000 overall CMC records, the rest of which had primary diagnoses of physical illness. This demonstrates that there may still be a considerable population of people with SMI who have not engaged with advance planning on CMC, such as those with limited contact with healthcare services, who may have utilised it differently. We did not include stratified analysis of those records which included engagement with MyCMC. CMC plans also contain other free text boxes for other data entry e.g. diagnosis and awareness of prognosis which were not analysed as part of this study.

Lastly, the sampling period includes the coronavirus pandemic and during this time there was heightened emphasis on the need for GPs to create ADM documents with people living with chronic illness who may be at higher risk of needing hospital admission. This may have increased the numbers of people with SMI who were actively offered the opportunity to make an ADM document and lowered the threshold for this offer. A limitation of the data available, however, was that it was not possible to identify the date of plan creation or completion, and so it was not possible to explore this possibility within the current dataset. Nonetheless it is of note that when there was higher policy pressure to create ADM documents a digital tool was considered feasible with this population. This avenue warrants further investigation to effectively assess the effect of the coronavirus pandemic on ADM document creation for those with SMI.

## Conclusions

To our knowledge this is the first study exploring the use of digital ADM documents for people with SMI in palliative care. The most surprising result is that this tool is being used by people with SMI but often not to plan for palliative or end of life care. Instead, this cohort and their supporting professionals, particularly GPs, have utilised a digital tool to plan for longer term physical and mental healthcare. Specific recommendations for future research and policy are outlined below:

- Digital tools tailored to support service users with SMI to plan for palliative care, physical and mental healthcare should be developed.
- In England and Wales there is an urgent need for policy makers to address the need for a digital ‘Advance Choice Document’ ahead of MHA reforms.
- These digital tools should utilise structured templates around patient preference to support inclusion of patient voice in ADM documents.
- These tools should have interoperability tailored for mental health scenarios e.g. accessible to paramedics and A&E staff as well as to mental health trust staff and Approved Mental Health Professionals (for use during MHA assessments).
- Training should be developed on the use of these digital tools to create and access ADM documents for health professionals including GPs. This training should emphasise an orientation towards a document which is service user owned and created.
- Future research should evaluate the uptake and use of these digital ADM documents.
- A particular focus should be exploring barriers and facilitators to uptake and use amongst those with intersectional vulnerabilities.

## Data Availability

Restrictions apply to the availability of these data, which were used under a special licence for this study under a data-sharing agreement with The Royal Marsden NHS Foundation Trust. Specific enquiries about the data can be made to Dr. Joanne Droney (joanne.droney@rmh.nhs.uk) upon reasonable request, with the permission of The Royal Marsden NHS Foundation Trust.
